# Honokiol suppresses metastasis of renal cell carcinoma by targeting KISS1/KISS1R signaling

**DOI:** 10.3892/ijo.2015.2950

**Published:** 2015-04-02

**Authors:** SHUJIE CHENG, VICTOR CASTILLO, ISAAC ELIAZ, DANIEL SLIVA

**Affiliations:** 1 Cancer Research Laboratory, Methodist Research Institute, Indiana University Health, Indianapolis, IN, USA; 2 Amitabha Medical Clinic and Healing Center, Santa Rosa, CA, USA; 3 Department of Medicine, School of Medicine, Indiana University, Indianapolis, IN, USA

**Keywords:** honokiol, renal cell carcinoma, metastasis, KISS1, KISS1R

## Abstract

Renal cell carcinoma (RCC) is a common urological cancer worldwide and is known to have a high risk of metastasis, which is considered responsible for more than 90% of cancer associated deaths. Honokiol is a small-molecule biphenol isolated from *Magnolia* spp. bark and has been shown to be a potential anticancer agent involved in multiple facets of signal transduction. In this study, we demonstrated that honokiol inhibited the invasion and colony formation of highly metastatic RCC cell line 786-0 in a dose-dependent manner. DNA-microarray data showed the significant upregulation of metastasis-suppressor gene *KISS1* and its receptor, *KISS1R*. The upregulation was confirmed by qRT-PCR analysis. Overexpression of KISS1 and KISS1R was detected by western blotting at the translation level as well. Of note, the decreased invasive and colonized capacities were reversed by *KISS1* knockdown. Taken together, the results first indicate that activation of KISS1/KISS1R signaling by honokiol suppresses multistep process of metastasis, including invasion and colony formation, in RCC cells 786-0. Honokiol may be considered as a natural agent against RCC metastasis.

## Introduction

Metastasis is the tendency of cancer cells to spread to distant organs in the body, which is considered responsible for more than 90% of cancer-associated deaths (1–4). It involves a multistep process including migration from the primary tumors, invasion to surrounding tissues, and proliferation leading to the colonization at distant sites (1,4). Accordingly, 25–30% of patients with renal cell carcinoma (RCC) have metastatic spread by the time they are diagnosed (5–7) and in these cases, the 5-year survival rate of patients is <10% (8,9). Moreover, 20–25% of suffers remain unresponsive to all treatments and the disease progresses rapidly (10,11). Honokiol, a small-molecule biphenolic compound isolated from *Magnolia* spp. Bark, has been shown to exhibit anticancer effects in different cancer types (12–17). The most widely investigated mechanism of its anticancer activities is apoptosis, which is induced *in vitro* and *in vivo* through multiple facets of signal transduction (12,14,16–25). Recently, several studies demonstrated that honokiol could also inhibit metastasis of breast, brain, gastric, lung and prostate cancer cells (13,21,26–32). However, only one study shows the metastasis suppression of RCC cells A-498 by honokiol through reversing epithelial-mesenchymal transition and blocking cancer stem cell properties (33). Definitely, there are other important targets involved in the process of RCC metastasis suppression by honokiol.

In this study, we found that honokiol inhibits the invasion and colony formation of highly metastatic RCC cells 786-0 (34) in a dose-dependent manner. DNA-microarray data showed significant upregulation of metastasis-suppressor gene *KISS1* and its receptor, *KISS1R*. Both of the upregulation were confirmed by qRT-PCR analysis. Overexpression levels of KISS1 and KISS1R were detected by western blotting at the translation level as well. Of note, inhibition of invasion and colony formation were reversed by *KISS1* knockdown. Taken together, our results indicate that honokiol suppresses the multistep process of metastasis, including invasion and colony formation, in RCC cells 786-0 via stimulation of KISS1/KISS1R signaling pathway.

## Materials and methods

### Cell culture and reagents

Human RCC cells 786-0 were obtained from ATCC (Manassas, VA, USA). Cancer cells were maintained according to the ATCC procedures. Honokiol (98%) (HonoPure^®^) was provided by Econugenics Inc. (Santa Rosa, CA, USA) and dissolved in DMSO at a concentration of 80 mM then stored at −20°C. DMSO was purchased from Sigma (St. Louis, MO, USA). Anti-KISS1, anti-KISS1R and anti-β-actin antibodies were obtained from Santa Cruz Biotechnology (Santa Cruz, CA, USA).

### Cell invasion assay

Cell invasion of 786-0 cells treated with honokiol (0–20 μM) was performed as previously described (35). Data points represent the mean ± SD of three individual filters within one representative experiment repeated at least twice.

### Colony formation assay

Colony formation of the 786-0 cells incubated in the presence of honokiol (0–40 μM) was evaluated as previously described (36). Data points represent the mean ± SD in one representative experiment repeated at least twice.

### DNA-microarray and quantitative RT-PCR analysis

The 786-0 cells were treated with honokiol (0, 40 μM) for 24 h and TaqMan^®^ Array Human Tumor metastasis was performed as previously described (37). In qRT-PCR analysis, the 786-0 cells were treated with honokiol (0–40 μM) for 24 h. Isolation, quantification, reverse transcription of RNA and PCR were performed as previously described (37). Relative quantity (RQ) of gene expression was normalized to β-actin and performed using the 2^−ΔΔCt^ method (38).

### Western blot analysis

The 786-0 cells were treated with honokiol (0–40 μM) for 24 h. Whole protein extracts isolated from cells were prepared and western blot analysis with KISS1 and KISS1R antibodies were performed as previously described (39). Western blots were quantified with HP-Scanjet 550c and analyzed by UN-SCAN-IT software (Silk Scientific, Orem, UT, USA).

### siRNA transfection

The 786-0 cells were transfected with human *KISS1* siRNA or control siRNA-A as previously described (37). After 48 h of transfection, the cells were harvested and *KISS1* knockdown was verified by western blot analysis.

### Statistical analysis

All the statistical analysis was performed using SigmaPlot 11.2.0 (Systat Software Inc., San Jose, CA, USA). Data are presented as mean ± SD. Statistical comparisons were carried out using ANOVA with the significance level adjusted using the repeated t-tests with Bonferroni correction. P-value <0.05 was considered to be significant.

## Results

### Honokiol inhibits invasion and colony formation of highly metastatic RCC cells

**Figure 1:**
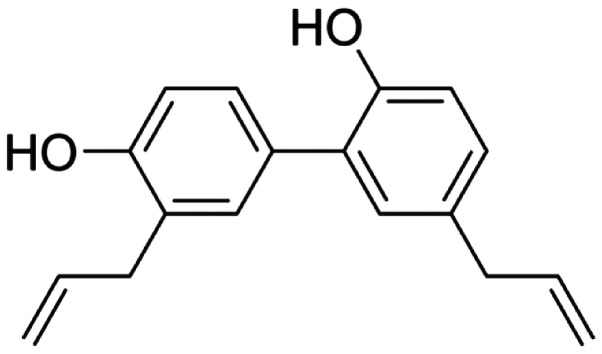
Structure of honokiol.

**Figure 2:**
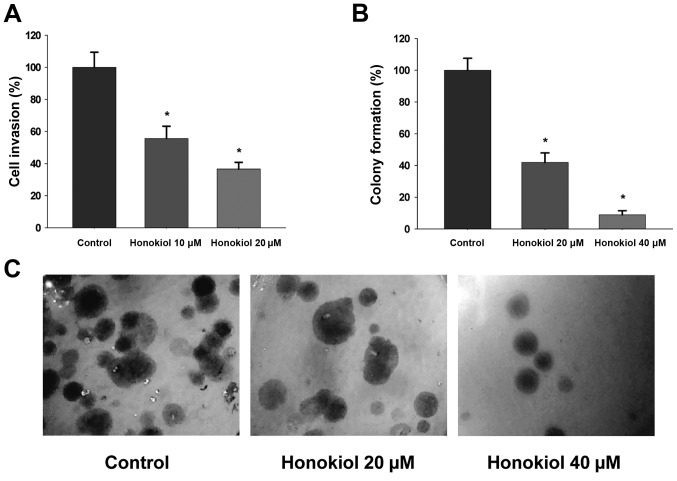
Effect of honokiol on the invasion and colony formation of the 786-0 cells. The 786-0 cells were treated with honokiol (A) (0–20 μM) or (B) (0–40 μM). Cell invasion through Matrigel and colony formation in agarose were determined as described in Materials and methods. Each bar represents the mean ± SD in one representative experiment repeated at least twice. Representative pictures of colony formation are shown (C). Statistical analysis by ANOVA, ^*^P<0.05.

To evaluate whether honokiol (Fig. 1) suppresses invasive behavior of highly metastatic RCC cells, the 786-0 cells were treated with honokiol (0–20 μM) for 24 h and cell invasion was determined as described in Materials and methods. As shown in Fig. 2A, honokiol inhibits cell invasion through Matrigel in a dose-dependent manner. Moreover, honokiol significantly decreases the number of anchorage-independent colonies formed, which is a key step in cancer metastasis (Fig. 2B and C). In summary, honokiol significantly inhibits invasion as well as colony formation of highly meta-static RCC 786-0 cells in a dose-dependent manner.

### Effect of honokiol on the expression of genes related to human tumor metastasis

In order to gain further mechanistic insight into the molecular events underlying metastasis inhibition of the 786-0 cells treated with honokiol, DNA-microarray analysis of 92 tumor metastasis-associated genes and 4 candidate endogenous control genes was performed. Table I summarizes the genes with large recurring expression differences compared with control. For example, significant upregulation was observed including the expression of metastasis suppressor gene (*KISS-1*, 28.56±11.17), genes encoding TIMP metalloproteinase inhibitor 4 (*TIMP4*, 14.25±4.04) and KISS-1 receptor (*KISS-1R*, 13.33±5.11). In addition, honokiol markedly suppresses expression of genes encoding chemokine (C-X-C motif) ligand 12 (CXCL12, 0.13±0.05), chemokine (C-C motif) ligand 7 (CCL7, 0.14±0.04), interleukin-18 (IL18, 0.23±0.05) and matrix metallo proteinase 7 (MMP7, 0.26±0.09).

### Honokiol activates KISS1/KISS1R signaling in highly meta-static RCC cells

**Figure 3:**
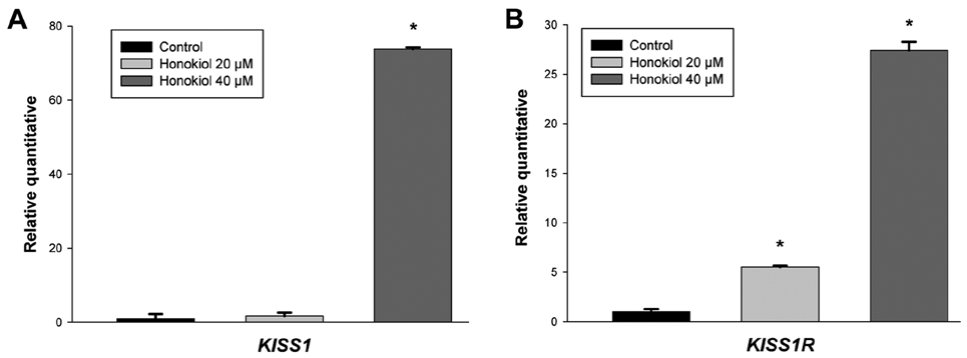
Honokiol stimulates mRNA expression of *KISS1* and *KISS1R* in the 786-0 cells. The 786-0 cells were treated with honokiol (0–40 μM) for 24 h and qRT-PCR analysis on *KISS1* and *KISS1R* were performed as described in Materials and methods. Each bar represents the mean ± SD of three independent experiments. Statistical analysis by ANOVA, ^*^P<0.05.

**Figure 4:**
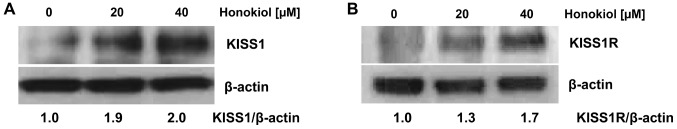
Honokiol induces expression of KISS1 and KISS1R in the 786-0 cells. The 786-0 cells were treated with honokiol (0–40 μM) for 24 h and the expression of KISS1 and KISS1R were evaluated by western blot analysis as described in Materials and methods. Representative results are shown. Similar results were obtained in at least two additional experiments.

Since recent studies showed that activation of KISS1/KISS1R signaling by kisspeptin treatment decreases the motility and invasive capacity of conventional RCC, and overexpression of KISS1 inhibits invasion of RCC cells Caki-1 (40,41), we confirmed the significant upregulation of *KISS1* and *KISS1R* in the 786-0 cells treated with honokiol by qRT-PCR (Fig. 3). In accordance with the change in mRNA, western blot analysis showed that honokiol stimulates expression of KISS1 and KISS1R in the 786-0 cells dose-dependently at the protein level (Fig. 4).

### Silencing KISS1 reverses suppression of invasion and colony formation

**Figure 5:**
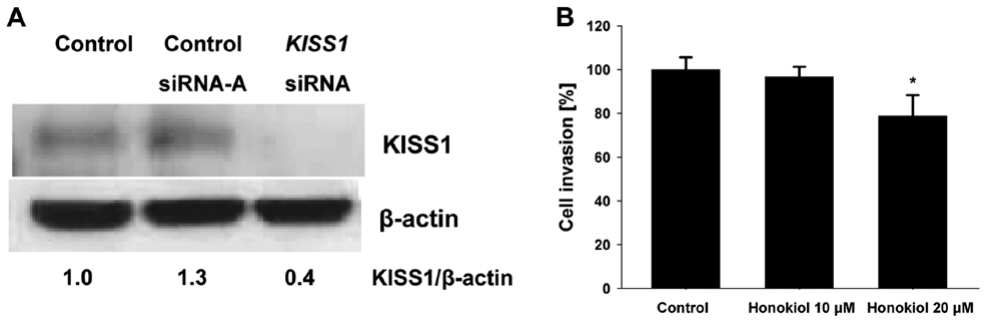
*KISS1* gene silencing partially rescues the effect of honokiol on cell invasion. The 786-0 cells were transfected with scrambled siRNA (siRNA-A) or *KISS1* siRNA as described in Materials and methods. (A) Western blot analysis of KISS1 was evaluated. (B) Invasion of the 786-0 cells through Matrigel was determined as described in Fig. 2A. Each bar represents the mean ± SD of three individual filters within one representative experiment repeated at least twice. Statistical analysis by ANOVA, ^*^P<0.05.

**Figure 6:**
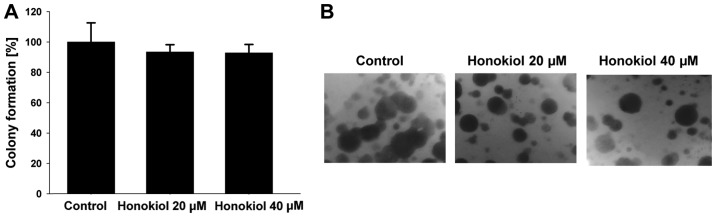
*KISS1* gene silencing reverses the effect of honokiol on colony formation. The 786-0 cells were transfected with scrambled siRNA (siRNA-A) or *KISS1* siRNA as described in Materials and methods. (A) Colony formation of the 786-0 cells in agarose was determined as described in Fig. 2B. (B) Representative images of colony formation are shown. Each bar represents the mean ± SD in one representative experiment repeated at least twice. Statistical analysis by ANOVA, ^*^P<0.05.

To determine whether the suppression of invasion and colony formation by honokiol are associated with the activation of KISS1/KISS1R signaling in the 786-0 cells, we silenced *KISS1* with siRNA as described in Materials and methods. As shown in Fig. 5, knockdown of *KISS1* partially rescues the effect of honokiol on cell invasion by more than 40%. Moreover, the effect of honokiol on colony formation of the 786-0 cells is markedly reversed by *KISS1* silencing (Fig. 6). These results further indicate that KISS1/KISS1R signaling is a major target of honokiol in suppressing metastasis of RCC cells.

## Discussion

In the present study, we investigated the role of honokiol in the metastasis of RCC cells. Our results showed that honokiol significantly inhibited the invasion and colony formation of highly metastatic RCC cells 786-0 in a dose-dependent manner. Moreover, honokiol markedly upregulated metastasis-suppressor gene *KISS1* and its receptor, *KISS1R*, at both transcription and translation levels. Interestingly, knockdown of *KISS1* partially rescued the effect of honokiol on cell invasion and its effect on colony formation of the 786-0 cells is reversed as well, indicating that KISS1/KISS1R signaling is a major target of honokiol in suppressing metastasis of RCC cells.

Metastasis suppressors are defined as molecules whose expression results in the suppression of metastasis processes and since 1986, more than 13 metastasis suppressors have been identified (42). The *KISS1* gene, initially discovered as a novel human malignant melanoma metastasis-suppressor gene (43), has been validated as an anti-metastatic gene by preclinical and clinical evidence in various types of cancer (44). The encoded KISS1 protein can be processed to a C-terminally amidated peptide termed metastin binding and activating the G-protein coupled receptor GPR54 (KISS1R) (45). Shoji *et al* found that metastin inhibited migration and invasion of RCC with overexpression of KISS1R (46). In addition, a recent study demonstrated that an absence of KISS1R expression was associated with rapid progression of conventional RCC in patients (40), suggesting KISS1/KISS1R signaling as a promising target in RCC.

Honokiol targets multiple signaling pathways such as nuclear factor κB (NF-κB), signal transducers and activator of transcription 3 (STAT3), mammalian target of rapamycin (mTOR) and epidermal growth factor receptor (EGFR), which have great relevance during cancer initiation and progression (47). Moreover, pharmacokinetic studies revealed that honokiol crossed the blood-brain barrier (BBB), the blood-cerebrospinal fluid barrier (BCSFB) and had a desirable bioavailability after intravenous administration in animal models (48) thus making it a suitable agent for clinical trials.

In summary, our results indicate that activation of KISS1/KISS1R signaling by honokiol decreases the invasiveness and colonized capacity of highly metastatic RCC cells. Furthermore, we confirmed that honokiol stimulated the expression of TIMP4 dose-dependently (data not shown). It is in accordance with the finding that metastin suppresses the motility and invasive ability of RCC cells which possess KISS1R through the downregulation of MMP-2 (49). As emerging studies show that KISS1R activates a series of signaling molecules such as protein kinase C (PKC), extra-cellular signal-regulated kinases 1 and 2 (ERK1/2), p38, and phosphatidylinositol-3-kinase (PI3K) (50), further studies are in progress to investigate the specific mechanism of honokiol, which may have the potential for use as a natural agent against RCC metastasis.

## Figures and Tables

**Table I:** Effect of honokiol on the expression of human tumor metastasis genes.

Gene	Description	RQ
*KISS1*	KISS-1 metastasis suppressor	28.56±11.17^a^
*TIMP4*	TIMP metalloproteinase inhibitor 4	14.25±4.04^a^
*KISS1R*	KISS1 receptor	13.33±5.11^a^
*TP53*	P53 tumor suppressor	2.24±0.16
*CXCL12*	Chemokine (C-X-C motif) ligand 12	0.13±0.05
*CCL7*	Chemokine (C-C motif) ligand 7	0.14±0.04
*IL18*	Interleukin-18	0.23±0.05
*MMP7*	Matrix metalloproteinase 7	0.26±0.09
*VEGFC*	Vascular endothelial growth factor C	0.42±0.04
*FGFR4*	Fibroblast growth factor receptor 4	0.53±0.04

DNA-microarray analysis was performed on TaqMan^®^ Array Human Tumor Metastasis as described in Materials and methods. The 786-0 cells were treated with honokiol (0 and 40 μM) for 24 h. Data are the means ± SD of three independent experiments. Analysis of the relative quantity gene expression (RQ) data was performed using the 2^−ΔΔCT^ method. Statistical analysis by ANOVA,

a P<0.05.
